# Abnormal Phenylalanine Metabolism of *Procapra przewalskii* in Chronic Selenosis in Selenium-Enriched Habitats

**DOI:** 10.3390/metabo13090982

**Published:** 2023-08-30

**Authors:** Hong Ren, Ping Zhou, Xiaoyun Shen

**Affiliations:** 1 North Sichuan Medical College, Nanchong 637100, China; renhong2017016@nsmc.edu.cn; 2 State Key Laboratory of Sheep Genetic Improvement and Healthy Production, Xinjiang Academy of Agricultural and Reclamation Sciences, Shihezi 832000, China; zhpxqf@163.com; 3 School of Life Science and Engineering, Southwest University of Science and Technology, Mianyang 621010, China; 4 World Bank Poverty Alleviation Project Office in Guizhou, Guiyang 550004, China

**Keywords:** *P. przewalskii*, phenylalanine, chronic selenosis, proteomics, metabolomics, biochemical analysis

## Abstract

Selenium (Se)-enriched habitats have led to chronic selenosis, seriously affecting the health and survival of Procapra przewalskii (*P*. *przewalskii*). Our targets were to explore the molecular mechanisms of chronic selenosis and to look for a new way to protect endangered species. The mineral contents of soils, grass, blood, and muscle were analyzed. The biochemical indices, antioxidant capability, and immune function were also investigated. The analyses of proteomics and metabolomics were also carried out. The results showed that the Se contents in the muscle and blood of *P*. *przewalskii*, and the soil and grass in the Se-enriched habitats were significantly higher than those in healthy pastures. The *P*. *przewalskii* in the Se-enriched habitats showed symptoms of anemia, decreased antioxidant capability, and low immune function. A total of 44 differential proteins and 36 differential metabolites were screened by analyzing their proteomics and metabolomics. These differential proteins and metabolites were involved in glycolysis pathway, amino acid biosynthesis, carbon metabolism, phenylalanine metabolism, and energy metabolism. In particular, phenylalanine metabolism was the common pathway of proteomics and metabolomics, which was an important finding in studying the mechanism of chronic selenosis in animals. This study will help us to further understand the mechanism of chronic selenosis in *P*. *przewalskii*, and it provides a scientific basis for the protection of endangered species in Se-enriched habitats.

## 1. Introduction

The Qinghai Lake Basin is the only natural habitat for Procapra przewalskii (*P*. *przewalskii*), with the coexistence of selenium (Se)-deficient ranches and Se-rich habitats [1]. *P*. *przewalskii* is the most endangered artiodactyl on the Tibetan Plateau and is mainly distributed on the ranches near the Qinghai Lake in China [1]. In recent years, many protection measures of rangelands have strictly limited their foraging range. The balancing mechanisms of mineral nutrition have been seriously damaged. Animals on Se-deficient ranches are incapable of foraging for Se-enriched plants in all seasons, resulting in Se deficiencies. *P*. *przewalskii* in a Se-enriched habitat are fed Se-enriched forage for a long time, resulting in chronic selenosis [2]. In addition, the use of Se-containing wastewater from industrial pollution for pasture irrigation will also lead to the passive storage of Se in plants; therefore, chronic selenosis might also be found in grazing animals in Se-polluted ranches [3]. Problems may also arise from the careless administration of Se supplements. As a result, accidental acute and chronic problems have become more common [4,5,6]. Se is necessary for animals and has various biological functions, such as strengthening their antioxidant defense, increasing their growth rate, improving reproductive performance, enhancing immunity, and decreasing the occurrence of diseases [7]. Se is also the most toxic essential element for animals. It was generally believed that 1–6 mg/kg of Se in soil and 3–4 mg/kg of Se in feed might cause chronic selenosis [8]. As Se poisoning is incurable, it has brought about severe challenges in terms of survival [9]. The symptoms of chronic selenosis in *P*. *przewalskii* are indigestion, weight loss, anemia, unresponsiveness, and a lack of vitality [10]. In addition, it can also affect fetal development, cause fetal malformations, and increase the mortality of neonatal animals. Samples of the liver, kidney, heart, spleen, lung, lymph nodes, pancreas, and brain also show lesions of degeneration, necrosis, nuclear deformation, telangiectasia, hyperemia, and hemorrhage in pathological examinations [11,12]. Chronic selenosis has brought great harm to the health of *P*. *przewalskii* and has seriously hindered the protection of other endangered species in the Qinghai Lake Basin [13,14]. However, so far, few researchers have studied chronic selenosis in *P*. *przewalskii*, especially using a combination of serology, proteomics, and metabolomics for exploring the mechanisms of chronic selenosis [15]. 

The purpose of this study was to explore the molecular mechanisms of chronic selenosis in *P*. *przewalskii* and look for a new way to protect endangered species in Se-enriched habitats. It also provides a reference for the study and prevention of chronic selenosis.

## 2. Materials and Methods

### 2.1. Geographical Environment

**Figure 1:**
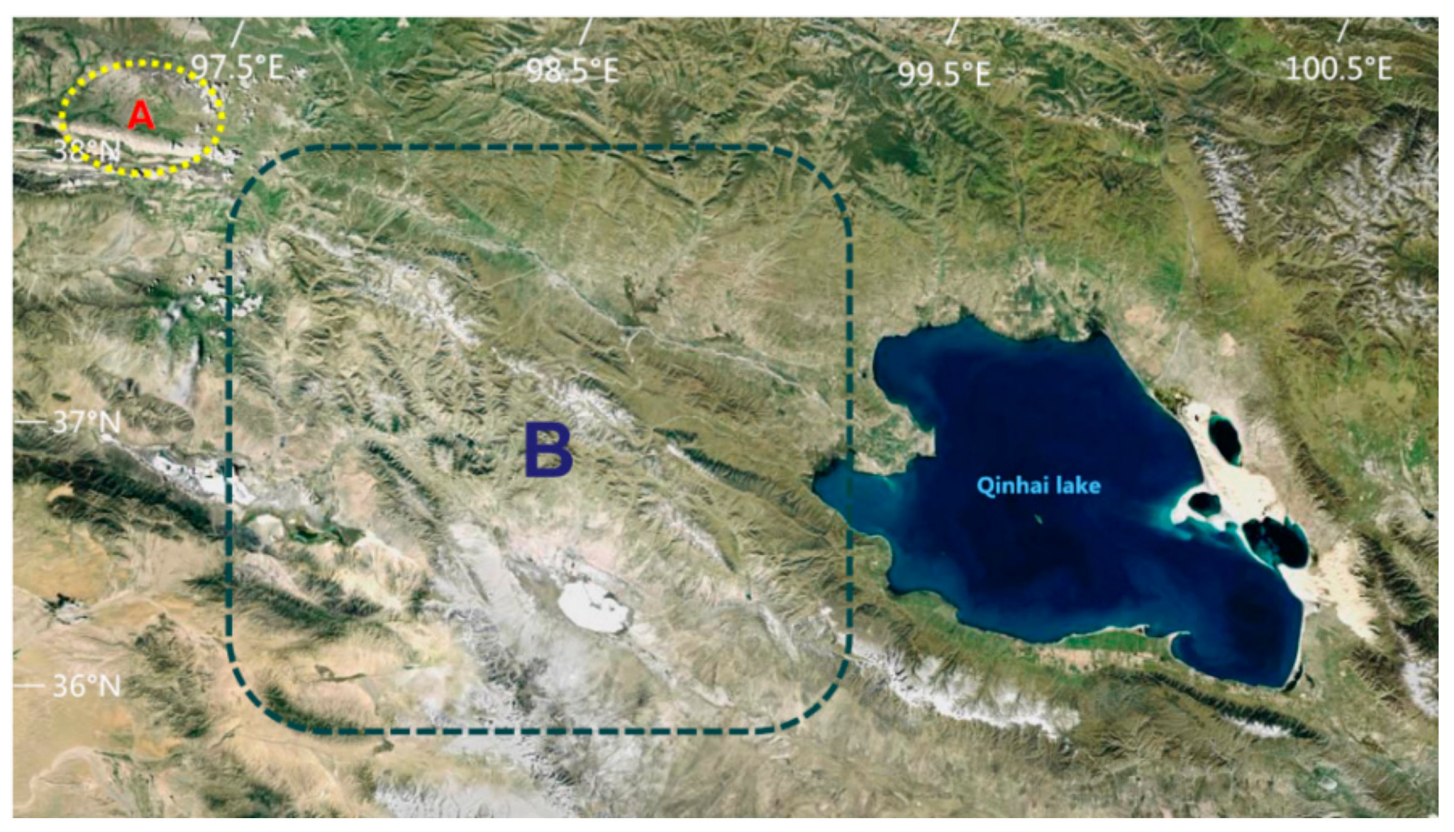
*P*. *przewalskii* habitats. A represents a Se-enriched habitat. B represents a healthy habitat.

The habitat of *P*. *przewalskii* is located on the Qinghai Lake basin, which has a plateau continental climate. It has strong sunshine, dry and windy conditions, short warm seasons, long cold seasons, and low rainfall (annual rainfall of 778 mm). Their Se-enriched habitats are located in the natural pasture of Shengge Township, Tianjun County, China (N: 38°53′23″–39°33′21″, E: 96°49′42″–97°31′48″). The healthy habitat belongs to the Bird Island Reserve in the ranches near the Qinghai Lake in China (N: 36°28′42″–38°25′53″, E: 97°51′23″–99°27′35″) (Figure 1). The vegetation is mainly *Roegneria nutans* (*Keng*) *Keng*, *Leymus secalinus*, *Carex mooscroftii*, *Artemisia frigida Willd*, *Elymus dahuricus Turcz*, *Artemisia frigida*, *Taraxacum mongolicum Hand.-Mazz*, *Carex stenophylla*, *Dactylis glomerata L*, *Stipa purpurea*, *Poa pratensis*, *Blysmus sinocompressus*, *Elymus nutans*, and *Agropyron cristatum.*

### 2.2. Sample Collection and Analysis

The samples of soil and plants from the ranches and blood and muscle from *P*. *przewalskii* included in this study were collected in August 2022. The soil samples were collected using a 30 mm diameter cylindrical core in the surface layer (0–10 cm). Four soil samples, each separated by 600 m, were bulked and placed in polythene bags. The soil sample was ground into a powder and filtered through a 10 mm screen. In order to avoid contamination of the grass samples, they were collected from 1 to 2 cm above the ground. According to previous experience, the researcher hid approximately 10–15 m away from the animals at 17–20 o’clock in July and used a tranquilizer gun (model: L50, China) with ketamine hydrochloride (SINICS approval: H35020148) as an anesthetic. Then, the anesthetic was injected into the animals (30 mg/time, 5 times within 3 min), and blood and muscle samples were collected. The samples of muscle and blood were stored in plastic test tubes that did not contain trace minerals. The blood was obtained from the jugular veins and the muscle was skeletal muscle obtained from the thigh. The muscle was extracted by penetrating the inside of the thigh skeletal muscle tissue with a puncture needle [16]. Approximately 20 min later, the animals returned to normal. The blood and muscle samples were subsequently transported to the laboratory for further analysis. 

These samples of soil, grass, blood, and muscle were dried and crushed in a vacuum freeze dryer (GIPP-5000FDA) at −80 °C. Samples of 0.2–1.0 g were dissolved using microwave digestion apparatus (Apole technology group Co., Ltd., Chengdu, China). The soil samples were dissolved by adding HF, HNO_3_, and HClO_4_ (5:2:5), and the samples of plant, blood, and muscle were dissolved by adding HNO_3_ and HClO_4_ (4:1). The concentrations of copper (Cu), iron (Fe), zinc (Zn), manganese (Mn), selenium (Se), and molybdenum (Mo) in the soil, grass, blood, and muscle samples were determined using an atomic absorption spectrophotometer (AA-7000, Shimadzu Co., Ltd., Tokyo, Japan). The results were then compared with certified elemental concentrations from the International Atomic Energy Agency and the National Institute of Standards standard substances to verify the accuracy of the results. The limited accuracy of the samples with very low contents resulted in concentrations below a particular threshold being recorded as containing trace amounts, as measurements of zero are difficult to prove.

### 2.3. Biochemical Tests on Blood 

The blood samples were placed in a fully automated blood analyzer (BC2800 Vet, Mindray Biotechnology Co., Ltd., Shanghai, China) to determine the cell volume (PCV), red blood cell count (RBC), hemoglobin (Hb), white blood cell count (WBC), and platelet count (PLT). According to the detection results for Hb, RBC, and PCV, the mean corpuscular hemoglobin (MCH), mean corpuscular volume (MCV), and mean corpuscular hemoglobin concentration (MCHC) were calculated. The blood samples were tested for antioxidant and immune function indicators according to the procedure of the diagnostic kit (Jianchen Biotechnology Co., LTD., Xiamen, China). The antioxidant capacity indices included lipid peroxide (LPO), total antioxidant capacity (T-AOC), total nitric oxide synthase (TNOS), catalase (CAT), glutathione peroxidase (GSH-Px), nitric oxide (NO), superoxide dismutase (SOD), and malondialdehyde (MDA). The immune indices included immunoglobulin G (IgG), tumor necrosis factor-alpha (TNF-α), interleukin-2 (IL-2), immunoglobulin A (IgA), included interleukin (IL)-1β, immunoglobulin M (IgM), and interleukin 6 (IL-6).

### 2.4. Proteomic Analysis of Serum

#### 2.4.1. TMT Mark

The serum samples were ground into a powder in liquid nitrogen, and the protein was extracted using the SDT cracking method and quantified using the BCA method. Then, FASP was used for tryptic hydrolysis, and a C18 cartridge was used to desalinate the enzymolysis peptide, followed by quantification using OD280. The samples were labeled as 100 μg peptides from each serum according to the instructions of the TMT kit (Thermo Fisher Scientific, Waltham, MA, USA).

#### 2.4.2. SCX Chromatographic Fractionation

All the TMT-labeled peptides were mixed and graded using an AKTA Purifier 100. The buffer conditions were 10 mM KH_2_PO_4_ and pH 3.0, in which liquid A was 25% (acetonitrile) can and liquid B was 500 mM KCl. The sample was separated at a flow rate of 1 mL/min by flowing through a chromatographic column which was balanced with liquid A. The time gradients of the liquid phase were 0–25 min, 25–32 min, 32–42 min, 42–47 min, 47–52 min, and 52–60 min. The concentration gradients of the liquid phase were 0–10%, 10–20%, 20–45%, 45–100%, and 100%. The time gradient phase corresponded to the concentration gradient of the liquid. During the elution process, the absorbance (D) value at a wavelength of 214 nm was monitored, and elution components were collected every 1 min, lyophilized, and desalted using a C18 cartridge.

#### 2.4.3. LC–MS/MS Analysis

The peptides were injected into a nanometric electrospray source using an ultra-high-performance liquid-phase separation system for tandem mass spectrometry with a sample column (Thermo scientific EASY column, 100 μm*2 × cm, 5 μm, C18). A Q-Exactive mass spectrometer (Thermo Fisher) was used to detect and analyze the isolated peptides. The resolution for detecting complete peptides was set to 70,000 at 200 *m*/*z*. The time was 60 min. The automatic gain control target was 3 × 10^6^, the maximum IT was 10 milliseconds, and the dynamic exclusion time was 40.0 s. The structure of the peptide was identified by means of the ion information obtained using high-energy impact dissociation (HCD). The impact energy was set to 30 eV. The underfill ratio was 0.1%. The resolution for detecting ion fragments was set to 17,500 at 200 *m*/*z*.

#### 2.4.4. Protein Identification and Analysis

To search the corresponding database for raw mass spectrometry test files, the protein identification and quantitative analysis results were obtained from the Proteome Discoverer 1.4 (Table 1).

#### 2.4.5. Bioinformatics Analysis

OmicsBean software was used to carry out GO annotation, and KEGG pathway annotation was used for the target protein set (http://www.omicsbean.cn/ (accessed on 30 September 2022). The process can be divided into four steps: blast, mapping, annotation, and annotation augmentation. The Fisher exact test was used to compare the distribution of each GO analysis and KEGG pathway in the target protein set and the total protein set. Then, the enrichment analysis of the GO annotation and KEGG annotation was performed on the target protein set.

### 2.5. Metabolomics Analysis in Serum

#### 2.5.1. Sample Processing

The serum samples were ground into a powder with liquid nitrogen and added to 400 μL of a pre-cooled H_2_O/MeOH/ACN solution (2:4:4, *v*/*v*). These were then swirl-mixed at −20 °C for 1 h, then stratified at 14,000× *g* for 1200 s. The supernatant was vacuum-dried, and 100 μL of acetonitrile aqueous solution (1:1, *v*/*v*) was added for the mass spectrometry analysis. This was reconstituted, vortexed, and centrifuged at 14,000× *g* for 900 s.

#### 2.5.2. Chromatography and Mass Spectrometry

The chromatographic conditions were as follows: Waters ACQUITY UPLC BEH Amide Column (2.1 mm × 150 mm,.1.7 μm). The mobile phase A was an aqueous solution containing 0.1% formic acid, and the mobile phase B was an aqueous solution containing 5% acetonitrile. The flow rate of the mobile phase was 0.40 mL/min. The gradient elution procedure was as follows: 0–0.5 min, 5% B; 0.5–1.0 min, 5% B; 1.0–9.0 min, 5~100% B; 9.0–12.0 min, 100% B; and 12.0–15.0 min, 5% B. Under the positive and negative ion modes, the sample size was 2.5 μL. The samples were placed in a 4 °C automatic injector during the entire analysis process.

The mass spectrum conditions were as follows: the positive and negative ion patterns were detected using an electrospray ion source (ESI) and a Q-Exactive four-pole and electrostatic field orbital trap high-resolution mass spectrometer (Thermo Fisher Scientific). The spray voltage was set to ±3.5 kV, the mass spectrum collection range was 80~1200 *m*/*z*, the resolution was 17,500, and the ion source was HESI. The capillary voltage was 3500 V/3500 V (positive/negative ion mode), the capillary temperature was 300 °C, the internal temperature was 320 °C, the Sheath gas was 45 arb, the auxiliary gas was 10 arb, and the RF level of the S-Lens was 55. 

The application software SIMCA-P 14.1 (Umetrics, Umea, Sweden) was used for pattern recognition and data analysis. A multi-dimensional statistical analysis was performed after Pareto-scaling pretreatment.

### 2.6. Data Processing

The mineral content and biochemical indices of different groups were analyzed with an ANOVA, and the significance was verified through bilateral testing, with differences of 5% (*p* < 0.05) or 1% (*p* < 0.01). *p* < 0.05 was considered to represent a significant difference. *p* < 0.01 was considered to be highly statistically significant. The results are presented as the mean ± standard deviation. SPSS software and DPS software were used for data processing. 

Compound Discoverer 3.0 software was used to pre-process the original proteomics and metabolomics data, such as peak extraction, peak comparison, peak correction, and normalization. The quality matching and secondary spectrum matching of metabolites were accurately identified by searching the laboratory database and BioCyc, HMDB, Lipidmaps, HFMDBmetlin, and other databases. 

## 3. Results

### 3.1. Mineral Content Analysis

The mineral contents of the soil and plants are shown in Table 2. The Se contents of soil and grass in the Se-enriched habitat were significantly higher than those in the healthy habitat (*p* < 0.01). The Fe contents of the soils and forages in the Se-rich habitat were slightly lower than those in healthy pastures (*p* < 0.05). The mineral contents of the blood and muscle samples are shown in Table 3. The Se contents of the blood and muscle samples from the Se-enriched habitat were significantly higher than those in the healthy habitat (*p* < 0.01). The Fe contents of the blood and muscle samples from the Se-rich habitat were significantly lower than those from the healthy pastures (*p* < 0.05). 

### 3.2. Biochemical Indices in the Blood

The levels of Hb, MCV, PLT, RBC (*p* < 0.05), and PCV(*p* < 0.01) from the Se-enriched habitat were significantly lower than those from the healthy habitat. The WBC count from the Se-enriched habitat was significantly higher than that from the healthy habitat (*p* < 0.01, Table 4). The levels of serum GSH-PX, T-AOC, SOD, and CAT from the Se-enriched habitat were significantly lower than those from the healthy habitat (*p* < 0.01). The levels of serum NO, MDA, T-NOS (*p* < 0.01), and LPO (*p* < 0.05) from the Se-enriched habitat were significantly higher than those from the healthy habitat (Table 5). The levels of serum IL-1β, IL-2, TNF-α, IgA, IgG, IL-6 (*p* < 0.01), and IgM (*p* < 0.05) from the Se-enriched habitat were significantly lower than those from the healthy habitat (Table 6).

### 3.3. The Proteomic Results in Serum

**Figure 2:**
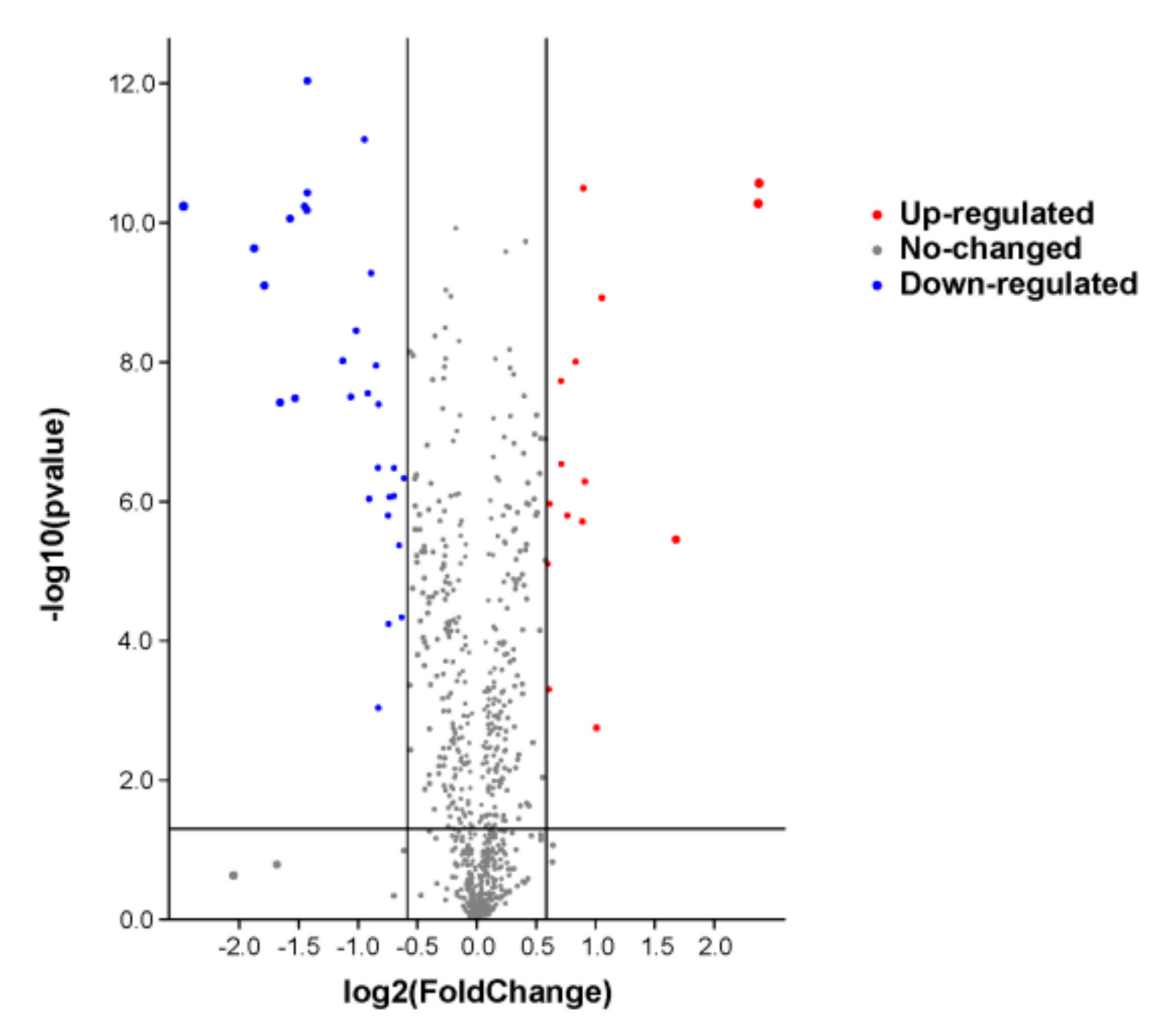
The differential protein volcano diagram. The abscissa is the multiple of difference, and the ordinate is the significant *p*-value of difference. The red dots are significantly upregulated proteins, the blue dots are significantly downregulated proteins, and the gray dots are proteins with no significant difference.

**Figure 3:**
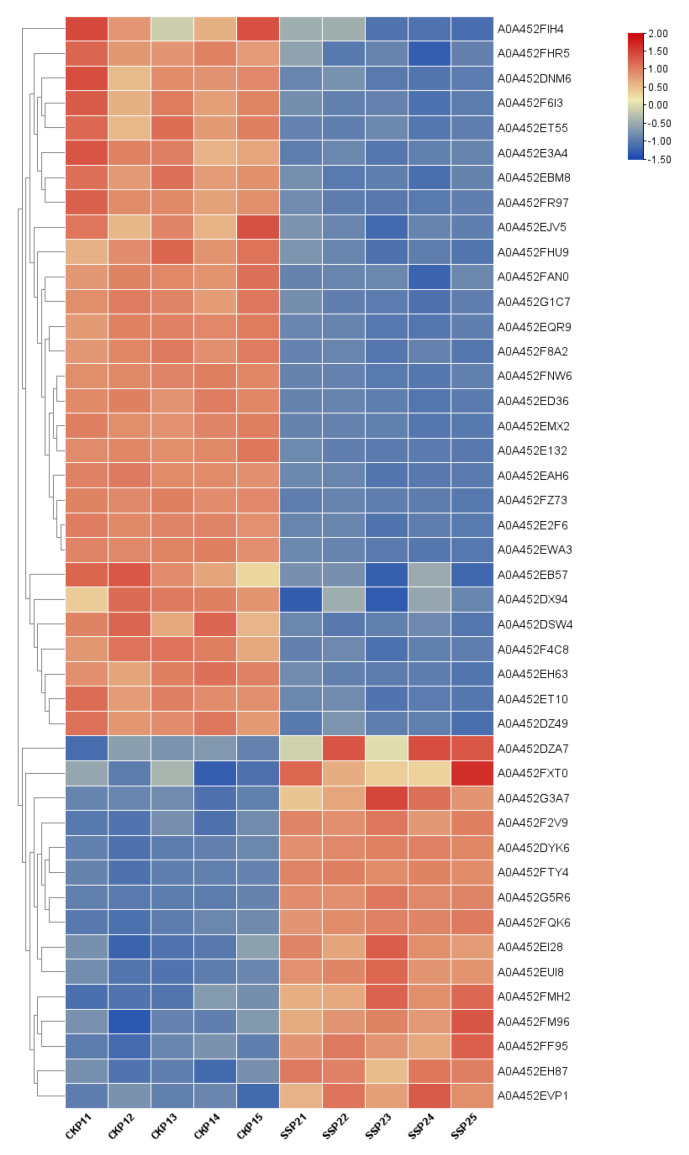
Results of cluster analysis of differentially expressed proteins. Each row represents a protein, and each column represents a group of samples. The logarithmic values of the expression amounts of significantly differentially expressed proteins in different samples are displayed in different colors in the heat map, with red representing a relatively high protein content and blue representing a relatively low protein content.

A total of 780 proteins and 2789 peptides were identified using quantitative proteomics technology and TMT labeling. The results of the volcanic map showed that there were significant differences between the two groups of sample data (Figure 2). The results of the cluster heat map showed that the differentially expressed proteins could be effectively distinguished from the comparison group, which further indicated the rationality of differentially expressed protein screening (Figure 3).

The differentially expressed proteins were screened according to the criteria of a fold change greater than 1.5 times (upregulation greater than 1.5 times or downregulation less than 0.667 times) and a *p*-value < 0.05. The results are shown in Table 7.

#### 3.3.1. Differential Protein Bioinformatics Analysis

##### GO Function Annotations

**Chart 1:**
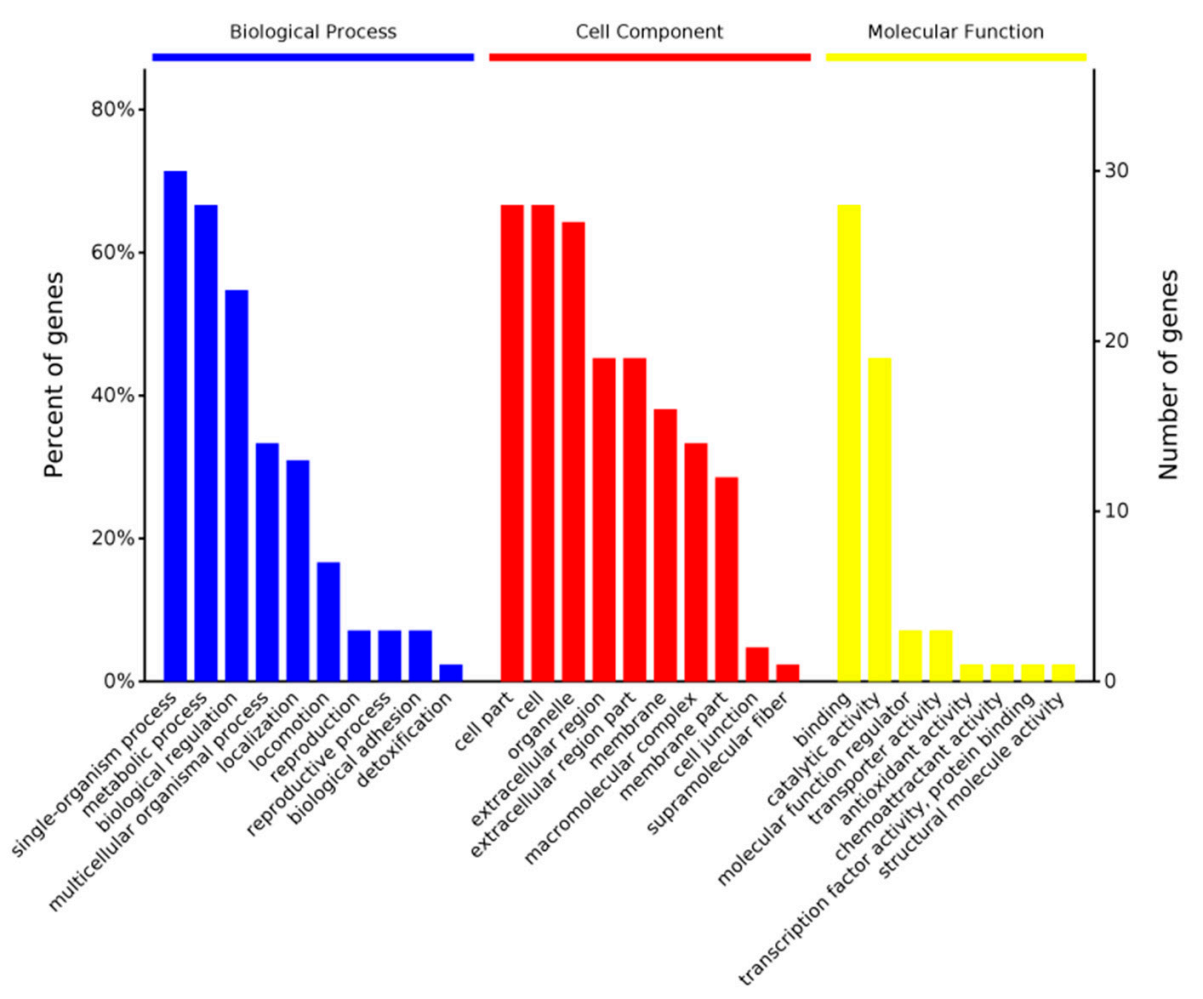
Differential protein GO (MF BP CC) functional annotation chart. The abscissa in the figure represents the enriched GO functional classification. The figure shows the top-ten level 2 entries in the enrichment analysis of three categories of BP, CC, and MF. The entries of each category are sorted from left to right according to their log value (*p*-value). The left side is more prominent. The vertical axis on the left represents the percentage of the number of proteins or genes corresponding to each entry to the total number of submitted proteins or genes. The vertical axis on the right represents the number of proteins or genes corresponding to each entry.

The software OmicsBean was used to annotate the target protein set. According to the classification of the biological process (BP), the target set was mainly involved in single-organism processes, metabolic processes, and biological regulation. According to the cellular component (CC), these differential proteins were mainly found in cell parts, cells, and organelles. Their molecular functions (MFs) mainly included binding and catalytic activity (Chart 1).

##### KEGG Pathway Annotations

**Chart 2:**
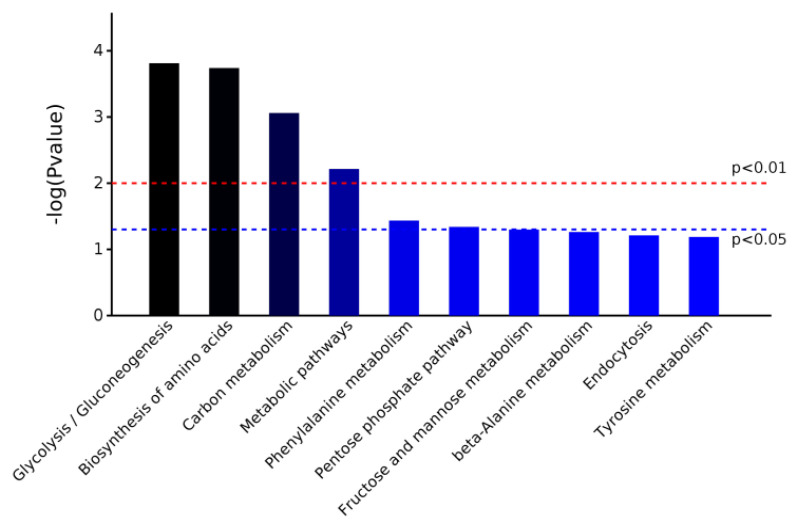
Pathway enrichment map of differentially expressed proteins based on KEGG metabolic pathway analysis.

The software OmicsBean was used to annotate the KEGG pathway for the target protein set. The KEGG metabolic pathway analysis showed that the differential proteins were enriched in five pathways: glycolysis/gluconeogenesis, biosynthesis of amino acids, carbon metabolism, metabolic pathways, and phenylalanine metabolism (Chart 2).

### 3.4. Metabolomics Results in Serum

**Figure 4:**
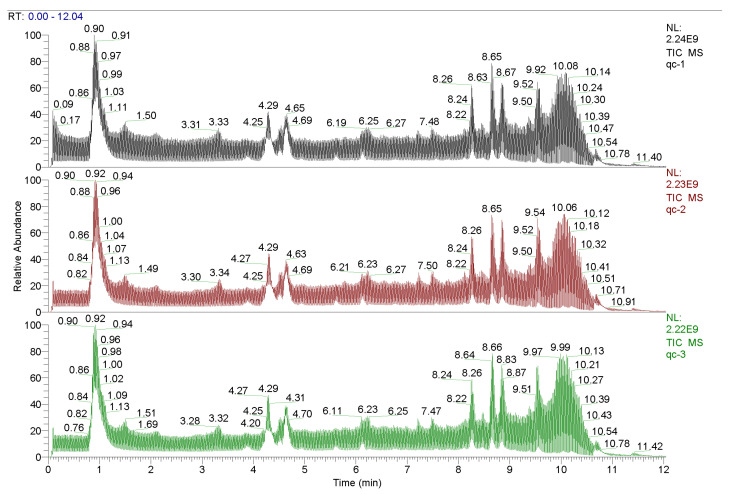
Total ionogram.

**Figure 5:**
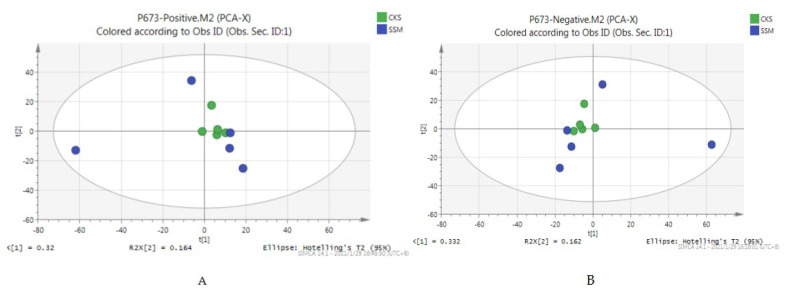
PCA scores of positive and negative ion modes. (**A**) the PCA score chart of the positive ion mode, and (**B**) the PCA score chart of the negative ion mode.

**Figure 6:**
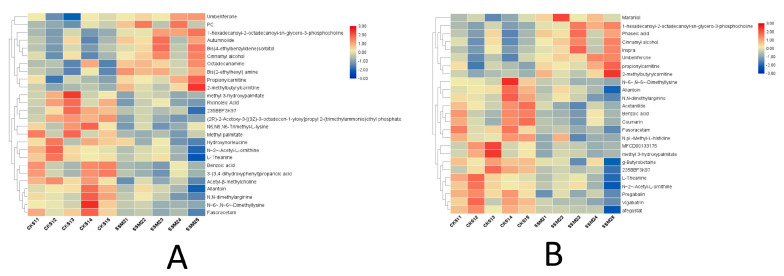
Results of the cluster analysis of differentially expressed metabolites. Each row represents a metabolite, and each column represents a group of samples. The logarithmic values of the expression amounts of significantly differentially expressed metabolites in different samples are displayed in different colors in the heat map, with red representing a relatively high metabolite content and blue representing a relatively low protein content. (**A**) the clustering heat map under the positive ion model, and (**B**) the clustering heat map under the negative ion model.

A total of 3246 metabolites were identified using LC–MS untargeted metabolomics technology. Thirty-six differentially expressed metabolites were screened according to the criteria of VIP ≥ 1 and *p*-value < 0.05. Among them, 13 proteins were upregulated and 23 proteins were downregulated (Table 8). The results of the total ion chromatogram showed that the response intensity and retention time of each chromatographic peak overlapped, indicating that the error was small throughout the entire experimental process (Figure 4). The results of the PCA analysis showed that the metabolic profiles of the two groups of samples had significant changes (Figure 5). The clustering heat map further confirmed this difference (Figure 6).

#### Differential Metabolite Bioinformatics Analysis

**Chart 3:**
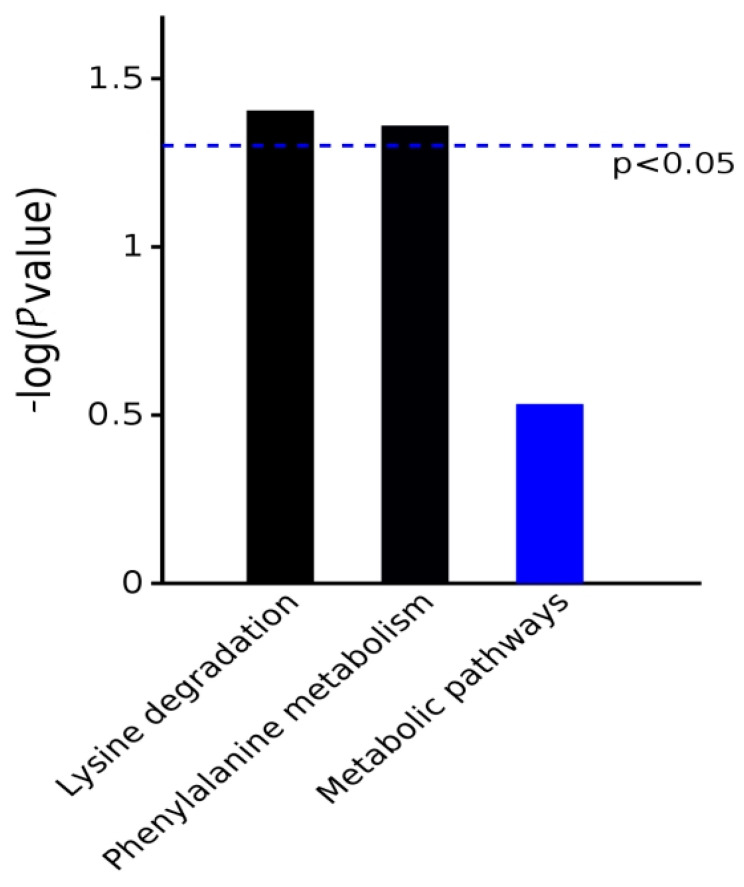
Pathway enrichment map of differentially expressed metabolites based on KEGG metabolic pathway analysis.

The differential metabolites obtained from the example group were submitted to the KEGG website for pathway analysis to obtain the metabolic pathways involved in the differential metabolites. The KEGG metabolic pathway analysis showed that the differential proteins were enriched in the two pathways of lysine degradation and phenylalanine metabolism (Chart 3).

## 4. Discussion

Natural weathering has led to an increase in the selenium content of rocks in some areas of China, the United States, Russia, and other regions. Domestic animals that lick these selenium-rich rocks can develop selenium poisoning [17,18]. For example, in the Qinghai Lake area of China, selenium-rich shale geological outcrops have led to selenium poisoning due to dry weather and high wind. Geochemical mapping can widely predict this risk [19,20]. The vegetation growing in Se-enriched soils also plays a part. When the content of available Se in the soil decreases or increases, it will directly affect the content of Se in plants. Plants are the main medium for transferring mineral elements in the soil to animals [21]. Therefore, the Se content in the soil will directly affect the nutrition and health of animals after being enriched by plants and transferred to animals [22]. Chronic selenosis in *P*. *przewalskii* is usually caused by consuming a large amount of Se-containing herbage for a long time, resulting in depression, dyspnea, abnormal gait, hair removal, hoof shedding, and other comprehensive symptoms [23,24]. Therefore, studying the effects of chronic selenosis on *P*. *przewalskii* is of great significance for protecting this endangered species.

In this study, we detected the Se content of soils and grass in a Se-enriched habitat and a healthy habitat. The detection data showed that the Se contents of the soil and grass samples from the Se-enriched habitat were significantly higher than those from the healthy habitat. We also examined the Se levels in blood and muscle samples taken from *P*. *przewalskii*, which again showed that the Se levels of the samples from the Se-rich pasture were significantly higher than those from the healthy habitat. Combined with the clinical symptoms of the animals in the Se-enriched habitat, it was confirmed that chronic selenosis has occurred in the Se-enriched habitat. The results of Se detection in the soil, grass, blood, and muscle samples were also consistent with the theory of Se mineral flow (soil, plant, and animal) [25]. 

The anemia index of animals can be reflected in the hematological parameters and blood Fe content. [26,27]. The results, in terms of the hematological parameters, showed that the contents of Hb, MCV, PLT, RBC, and PCV in the Se-enriched habitat in *P*. *przewalskii* were lower than those in the healthy habitat. In addition, the Fe levels in the blood and muscle samples from *P*. *przewalskii* in the Se-enriched habitat were significantly lower than those in the healthy habitat. These results showed that *P*. *przewalskii* had symptoms of anemia. Studies have shown that when the Se content in an organism is significantly elevated, the methyl transfer reaction is blocked, and the metabolism of VB_12_ and folate is abnormal. This leads to Fe deficiency and Fe-deficiency anemia [28]. In addition, the content of iron in soil and plants in selenium-rich habitats was significantly lower than that in healthy habitats, which may also be one of the causes of anemia in *P*. *przewalskii*. 

The body’s defense system includes the non-enzymatic system and enzymatic system. Non-enzymatic systems mainly include VE, VC, cysteine, GSH, Cu, Fe, Zn, and Se. The enzymatic system consists of SOD, GSH-Px, CAT, and other antioxidant enzymes. Antioxidants are important substances in the body’s defense system, and their main function is to stabilize and neutralize free radicals [29,30,31,32]. In addition, T-AOC and MDA reflect antioxidant functions, while NOS and NO are directly affected by antioxidant functions [33,34,35,36]. The results of antioxidant capacity in *P*. *przewalskii* showed that the levels of GSH-Px, T-AOC, SOD, and CAT in the Se-enriched habitat were significantly lower than those in the healthy habitat. The levels of NO, MDA, T-NOS, and LPO were significantly higher than those in the healthy habitat. These results indicated that Se-rich habitats will lead to antioxidant function problems, the oxidation system being out of balance, and serious oxidation dysfunction in the body. On the other hand, studies have shown that decreased GSH-Px activity in the blood leads to an increase in peroxides on membrane lipids, the destruction of the biological activity of hemoglobin, and a decrease in the resistance of red blood cells to the hypotonic environment, ultimately leading to chronic anemia [37,38,39]. This is consistent with the symptoms of anemia, as indicated by the biochemical results in the blood.

The levels of IL-1β, IL-2, TNF-α, IgA, IgG, IL-6, and IgM in the serum directly reflect the immune function of the organism [40]. Regarding the results of immune function, the levels of serum IL-1β, IL-2, TNF-α, IgA, IgG, IL-6, and IgM in the Se-enriched habitats were significantly lower than those in the healthy habitats. These results indicated that the immune function of *P*. *przewalskii* in the Se-enriched habitat was decreased. Some studies have reported that antioxidant function is closely related to immune function, and a reduction in the antioxidant function of an organism will lead to an increase in oxygen free radicals and others. In addition, the occurrence of the lipid peroxygenation reaction will cause peroxygenation damage to the body tissue, causing cell metabolism disorders and an immunity decline [41,42,43,44,45]. In this study, the detected immune and antioxidant indices in the serum confirmed that the changing trend in antioxidant and immune functions was consistent, which was also consistent with previous studies.

A total of 44 differential proteins and the 36 differential metabolites were screened using proteomics and metabolomics technology in the serum of *P*. *przewalskii*. Among them, 13 differential metabolites were upregulated, and the remaining 23 differential metabolites were downregulated. The selected proteins and metabolites were involved in glycolytic pathway disorders, amino acid biosynthesis, carbon metabolism, phenylalanine metabolism, and energy metabolism. It is particularly noteworthy that the results of the proteomic and metabolomic enriched pathways in this study mapped to a common pathway, namely, phenylalanine metabolism.

Phenylalanine is an essential amino acid, which is converted to tyrosine by phenylalanine hydroxylase [46]. Phenylalanine hydroxylase is an Fe enzyme. When the Fe in the organism is deficient, its activity decreases; therefore, the conversion of phenylalanine to tyrosine is blocked, resulting in an increase in serum phenylalanine levels. Studies have shown that the plasma phenylalanine levels in Fe-deficient mice increased significantly and decreased after treatment with Fe [47]. Abnormal phenylalanine metabolism can lead to anemia symptoms in an organism, which are consistent with the anemia symptoms found using serum biochemical indicators and Fe content.

Phenylalanine is also closely related to antioxidant function. Studies have found that phenylalanine can increase SOD in the plasma, protect the vascular endothelium from damage due to reactive oxygen species (the product of cellular REDOX), regulate the antioxidant function of the organism by regulating the removal of oxygen free radicals, and improve the secretion of endothelial cells [48,49]. In this study, we measured serum antioxidant function indices and found that the SOD in the Se-enriched habitat was significantly lower than in the healthy habitat. Combined with the analysis of other antioxidant indices, this showed that the antioxidant function was significantly decreased, indicating that abnormal metabolism of phenylalanine might be associated with decreased antioxidant function.

Phenylalanine metabolism affects immune function. Studies have shown that phenylalanine deficiencies can atrophy the thymus and spleen in animals, impairing the function of the mononuclear phagocytic system [50]. It has also been found that high-dose intraperitoneal injection of phenylalanine in mice can inhibit the antibody response, which might be related to the inhibition of the humoral immune function through phenylalanine metabolism, especially phenylethylamine [51]. The results in this study showed that immune function decreased, suggesting that these findings might be related to abnormal phenylalanine metabolism.

The main function of phenylalanine is to produce tyrosine, which acts on the nerves, liver, and other tissues [52]. Phenylalanine metabolism can also synthesize certain hormones; neurotransmitters such as DA, NE, E; and the production of melanin in the skin and adrenal medulla [53]. Therefore, stable phenylalanine metabolism at a reasonable level is an important condition for the normal growth, development, and physiological functioning of the body. [54,55,56]. Diseases of the liver and kidney, genetics, immunity, neurohumoral activation, and other factors can cause phenylalanine metabolism disorders, resulting in the obstruction of phenylalanine conversion to tyrosine and an increase in blood phenylalanine concentration or the phenylalanine/tyrosine ratio [57]. Studies have shown that long-term high levels of phenylalanine or a decline in the ratio of tyrosine to phenylalanine can cause nervous system damage, albinism, phenylketonuria, and other diseases [58,59]. In particular, damage to the nervous system can lead to intellectual developmental deficits, motor dysfunction, depression, mental disorders, developmental delays, coma, and even death [59,60]. Both chronic and acute selenosis can present varying degrees of neurological symptoms in *P*. *przewalskii*. Based on this analysis, we speculated that the neurological symptoms of selenosis might be related to abnormal phenylalanine metabolism.

Leptin is the expression product of obesity genes [61]. It is widely involved in the regulation of various physiological activities of the human body, such as food intake, energy consumption and metabolism, hormone regulation, immunity, and vascular proliferation [62]. The levels of leptin, as a negative feedback signal of central fat deposition in the brain, increase with increasing fat deposition [63,64]. A study found that human plasma leptin levels and phenylalanine concentrations are positively correlated [65]. Compared with healthy controls and patients with PKU on a strictly controlled diet (the content of phenylalanine in the diet was controlled below 0.19 mmo/L), the plasma leptin levels in patients with PKU without a controlled diet were significantly higher [66]. It has been suggested that disorders of phenylalanine metabolism can affect the occurrence of various diseases through leptin. Chronic selenosis in *P*. *przewalskii* resulted in symptoms such as weight loss, anemia, and lack of vitality, which may be related to abnormal phenylalanine metabolism, resulting in abnormal leptin levels [67,68,69].

Some studies have found that abnormal metabolism of phenylalanine can also lead to abnormal metabolism of vitamin B12 (VB_12_), thereby causing related diseases [70]. The main functions of VB_12_ include promoting the development and maturation of red blood cells, maintaining hematopoietic function in a normal state, preventing pernicious anemia, maintaining the health of the nervous system, promoting the metabolism of carbohydrates, promoting fat and protein synthesis, eliminating restlessness, and improving concentration. VB_12_ is also necessary for nervous system function [71,72]. Previously, we also analyzed the relationship between Se and VB_12_, indicating that excessive Se leads to abnormal metabolism of VB_12_ in organisms, leading to Fe-deficiency anemia. It has been suggested that Se enrichment in *P*. *przewalskii* may lead to abnormal phenylalanine metabolism. This may lead to abnormal metabolism of VB_12_ and eventually lead to Fe-deficiency anemia and neurological symptoms.

In addition, the liver is the main location for phenylalanine metabolism [73]. When liver diseases occur, such as cirrhosis and necrosis, the enzymes required for phenylalanine metabolism are destroyed, resulting in disorders of phenylalanine metabolism [74]. Studies have shown that the plasma phenylalanine concentration in patients with liver cirrhosis and liver cancer is at a high level, which is positively correlated with the removal of phenylalanine metabolism through alanine aminotransferase (reflecting the degree of liver damage) [75,76]. The liver is the main lesion site of chronic selenosis in *P*. *przewalskii*, which will cause expansion, rupture, hemorrhage of the central hepatic vein and hepatic sinusoids, and focal necrosis, revealing that abnormal phenylalanine metabolism is consistent with the pathological changes associated with Se poisoning in *P*. *przewalskii* [77,78]. 

The above analysis showed that abnormal phenylalanine metabolism may be one of the reasons for the symptoms of chronic selenosis in *P*. *przewalskii*, such as weight loss, anemia, a low antioxidant capacity, decreased immune function, lack of vitality, depression, and sluggishness.

In summary, the series of symptoms caused by abnormal phenylalanine metabolism coincides with the symptoms of chronic selenosis, which is an important finding for studying the mechanism of chronic selenosis in *P*. *przewalskii*. The exact mechanisms of abnormal phenylalanine metabolism caused by chronic selenosis in *P*. *przewalskii* remain to be verified by further experiments. We will address this topic in our next study.

## 5. Conclusions

In our study, we found that a Se-enriched habitat caused chronic selenosis in *P*. *przewalskii*. This was characterized by anemia, decreased antioxidant function, decreased immune function, and abnormal phenylalanine metabolism. The findings of this study will help to improve the understanding of the mechanism of chronic selenosis in *P*. *przewalskii* and provide a scientific basis for the protection of endangered species in relation to excessive Se in natural habitats.

## Figures and Tables

**Table 1:** Proteome Discoverer parameters used for searching the database.

Item	Value
Type of search	MS/MS ion search
Enzyme	Trypsin
Mass values	Monoisotopic
Max missed cleavages	2
Fixed modifications	Carbamidomethyl (C), TMT 10 plex (N-term), TMT 10 plex (K)
Variable modifications	Oxidation (M), TMT 10 plex (Y)
Peptide mass tolerance	±20 ppm
Fragment mass tolerance	0.1 Da
Protein mass	Unrestricted
Database	Uniprot
Database pattern	Decoy
FDR	≤0.01
Protein quantification	The protein ratios are calculated as the median of only the unique peptides of the protein
Experimental bias	Normalizes all peptide ratios by the median protein ratio. The median protein ratio should be 1 after the normalization.

**Table 2:** Mineral contents in the soil and grass (μg/g).

Elements	Soil		Grass	
	Se-Rich Habitat	Healthy Habitat	Se-Rich Habitat	Healthy Habitat
Copper (Cu)	5.24 ± 1.36	5.82 ± 1.68	3.69 ± 1.01	4.03 ± 0.79
Manganese (Mn)	274.63 ± 35.69	266.84 ± 28.94	71.54 ± 11.58	68.74 ± 13.68
Iron (Fe)	7681.89 ± 741.52 *	7985.51 ± 661.21	699.73 ± 197.13 *	714.58 ± 187.95
Zinc (Zn)	44.84 ±7.51	43.11 ± 6.85	31.21 ± 8.04	33.57 ± 9.11
Selenium (Se)	2.82 ± 0.043 **	0.67 ± 0.022	3.39 ± 0.059 **	0.89 ± 0.021
Molybdenum (Mo)	1.78 ± 0.31	1.86 ± 0.28	1.57 ± 0.22	1.96 ± 0.67

* At the level of *p* < 0.05, ** at the level of *p* < 0.01.

**Table 3:** Mineral contents in the blood and muscle.

Elements	Blood (μg/mL)		Muscle (μg/g)	
	Se-Rich Habitat	Healthy Habitat	Se-Rich Habitat	Healthy Habitat
Cu	0.84 ± 0.17	0.91 ± 0.27	35 ± 1.04	39 ± 1.17
Mn	0.49 ± 0.057	0.51 ± 0.033	6.17 ± 0.19	5.98 ± 0.16
Fe	381.35 ± 56.87 **	519.45 ± 49.76	141.75 ± 18.28 **	189.65 ± 20.41
Zn	7.79 ± 1.68	7.48 ± 1.72	95.84 ± 3.47	87.65 ± 3.77
Se	0.54 ± 0.021 **	0.11 ± 0.017	1.16 ± 0.05 **	0.35 ± 0.03
Mo	0.29 ± 0.016	0.31 ± 0.023	0.98 ± 0.03	1.07 ± 0.04

** at the level of *p* < 0.01.

**Table 4:** Results of hematological tests.

Hematological Parameters	Blood	
	Se-Rich Habitat	Healthy Habitat
Hb (g/L)	145.48 ± 5.25 *	178.94 ± 7.94
RBC (×10^12^/L)	10.02 ± 0.26 *	12.04 ± 0.24
PCV (%)	38.17 ± 1.77 **	51.87 ± 1.54
MCV (fl)	38.09 ± 0.66 *	43.08 ± 0.68
MCH (pg)	14.52 ± 0.38	14.86 ± 0.24
MCHC (g/L)	381.14 ± 14.61	344.98 ± 10.54 *
WBC (10^9^/L)	18.67 ± 0.42	11.37 ± 0.84 **
PLT (×10^9^/L)	441.74 ± 13.87 *	468.85 ± 21.98

* At the level of *p* < 0.05, ** at the level of *p* < 0.01.

**Table 5:** Results of antioxidant indices.

Antioxidant Indices	Serum	
	Se-Rich Habitat	Healthy Habitat
GSH-Px (U/mL)	46.84 ± 3.98 **	69.54 ± 6.61
SOD (U/mL)	49.86 ± 7.69 **	67.85 ± 13.54
CAT (U/mL)	3.13 ± 0.54 **	6.11 ± 0.75
T-AOC (U/mL)	4.01 ± 0.41 **	7.88 ± 1.14
MDA (nmol/mL)	13.02 ± 3.11	4.44 ± 2.97 **
NOS (U/mL)	70.98 ±7.79	39.74 ± 6.83 **
NO (μmol/mL)	8.94 ± 2.14	4.39 ± 1.17 **
LPO (mmol/mL)	3.25 ± 0.97	2.11 ± 0.64 *

* At the level of *p* < 0.05, ** at the level of *p* < 0.01.

**Table 6:** Results of immune indices.

Immune Indices	Serum	
	Se-Rich Habitat	Healthy Habitat
IL-1β (ng/L)	42.98 ± 7.64 **	104.91 ± 10.28
IL-2 (ng/L)	174.65 ± 19.97 **	255.41 ± 22.68
IL-6 (ng/L)	296.15 ± 38.17 **	502.96 ± 55.47
TNF-α (ng/L)	523.63 ± 55.41 **	711.47 ± 79.51
IgA (g/L)	048 ± 0.066 **	1.02 ± 0.19
IgG (g/L)	5.99 ± 0.64 **	9.07 ± 0.64
IgM (g/L)	3.35 ± 0.17 *	4.11 ± 0.36

* At the level of *p* < 0.05, ** at the level of *p* < 0.01.

**Table 7:** The differential proteins screened.

Number	Proteins	Ratio
A0A452ED36	B-cell receptor-associated protein	0.180
A0A452EAH6	PDZ domain-containing protein	0.273
A0A452F8A2	GYF domain-containing protein	0.289
A0A452FR97	Phosphoglycerate mutase	0.317
A0A452E132	Phosphoglycerate kinase	0.336
A0A452F4C8	Exocyst complex component Sec8	0.346
A0A452EWA3	Non-specific serine/threonine protein kinase	0.366
A0A452E2F6	Alpha-1-acid glycoprotein	0.371
A0A452EMX2	Uncharacterized protein	0.372
A0A452FZ73	Creatine kinase	0.372
A0A452EH63	Fructose-bisphosphate aldolase	0.457
A0A452DZ49	Uncharacterized protein	0.479
A0A452G1C7	Uncharacterized protein	0.494
A0A452FNW6	Glyceraldehyde-3-phosphate dehydrogenase	0.519
A0A452FAN0	2-phospho-D-glycerate hydrolyase	0.530
A0A452FHR5	Uncharacterized protein	0.533
A0A452EQR9	RRM domain-containing protein	0.539
A0A452ET10	Uncharacterized protein	0.555
A0A452FHU9	Uncharacterized protein	0.562
A0A452FIH4	PITH-domain-containing protein	0.563
A0A452EBM8	Chloride channel protein	0.563
A0A452DNM6	Arp2/3 complex 34 kDa subunit	0.596
A0A452EB57	Proteasome subunit beta	0.597
A0A452DSW4	Amine oxidase	0.601
A0A452E3A4	Uncharacterized protein	0.617
A0A452ET55	Triosephosphate isomerase	0.617
A0A452EJV5	SAM domain-containing protein	0.635
A0A452DX94	Glutathione transferase	0.645
A0A452F6I3	Uncharacterized protein	0.653
A0A452G3A7	Uncharacterized protein	1.512
A0A452FXT0	DZF-domain-containing protein	1.525
A0A452EVP1	Apolipoprotein D	1.532
A0A452EUI8	Apolipoprotein C-II	1.635
A0A452FF95	Ig-like domain-containing protein	1.638
A0A452EI28	Uncharacterized protein	1.697
A0A452F2V9	V-type proton ATPase subunit C	1.782
A0A452FMH2	Serum amyloid A protein	1.852
A0A452FTY4	Serum amyloid A protein	1.863
A0A452EH87	Ig-like domain-containing protein	1.879
A0A452DZA7	Ig-like domain-containing protein	2.012
A0A452FQK6	Uncharacterized protein	2.075
A0A452FM96	Uncharacterized protein	3.199
A0A452DYK6	LRRcap domain-containing protein	5.170
A0A452G5R6	Haptoglobin	5.198

Number: the number of the differential proteins; Proteins: the name of the differential proteins; Ratio: the expression ratio of the differential protein compared with the control group (healthy habitat).

**Table 8:** The differential metabolites screened.

Number	Metabolites	Ratio
M1136	MFCD00133175	0.236674101
M1131	Methyl 3-hydroxypalmitate	0.245912491
M0096	Pregabalin	0.360438539
M0217	Vigabatrin	0.405421635
M0830	N~6~,N~6~-Dimethyllysine	0.462717965
M0518	L-Theanine	0.505753644
M0476	Fasoracetam	0.530718405
M1143	Coumarin	0.584921507
M0781	Afegostat	0.586163676
M0279	g-Butyrobetaine	0.598788438
M0333	Acetanilide	0.600654881
M0456	235BBF3K97	0.602385586
M0430	N.pi.-Methyl-L-histidine	0.654847868
M0168	Ricinoleic Acid	0.352162448
M0223	Benzoic acid	0.46156872
M0124	N~2~-Acetyl-L-ornithine	0.515348311
M0068	Hydroxynorleucine	0.589651438
M0075	3-(3,4-dihydroxyphenyl)propanoic acid	0.599884946
M0216	Allantoin	0.624748837
M0105	N,N-dimethylarginine	0.627098881
M0113	(2R)-2-Acetoxy-3-[(9Z)-9-octadecen-1-yloxy]propyl 2-(trimethylammonio)ethyl phosphate	0.673983498
M0121	N6,N6,N6-Trimethyl-L-lysine	0.802597421
M0189	Methyl palmitate	0.820344187
M0009	PC	1.240687574
M0306	Octadecanamine	1.415271685
M0019	Bis(4-ethylbenzylidene)sorbitol	1.432326828
M0324	Bis(2-ethylhexyl) amine	1.441261393
M0308	Cinnamyl alcohol	1.471295541
M0341	Autumnolide	1.523646777
M1020	Umbelliferone	1.075196303
M0481	Maraniol	1.189934366
M1275	1-hexadecanoyl-2-octadecanoyl-sn-glycero-3-phosphocholine	1.373282197
M0115	Inspra	1.527846994
M1220	Phaseic acid	1.548361001
M0339	Propionylcarnitine	1.756348021
M0422	2-methylbutyrylcarnitine	1.953783596

Number: the number of the differential metabolites; Metabolites: the name of the differential metabolites; Ratio: the expression ratio of the differential metabolite compared with the control group (healthy habitat).

## Data Availability

The datasets generated and analyzed in the current study are available from the corresponding author upon reasonable request. The data are not publicly available due to privacy.
